# Supplementary material for: FGR Src family kinase causes signaling and phenotypic shift mimicking retinoic acid-induced differentiation of leukemic cells
Source: Oncotarget. 2025 Mar 21;16:202–18. doi: 10.18632/oncotarget.28705 (PMC11927794; doi:10.18632/oncotarget.28705)
Supplement: Supplementary file 1 [file oncotarget-16-28705-s001.pdf]

FGR Src family kinase causes signaling and phenotypic shift mimicking retinoic acid-induced differentiation of leukemic cells

SUPPLEMENTARY MATERIALS

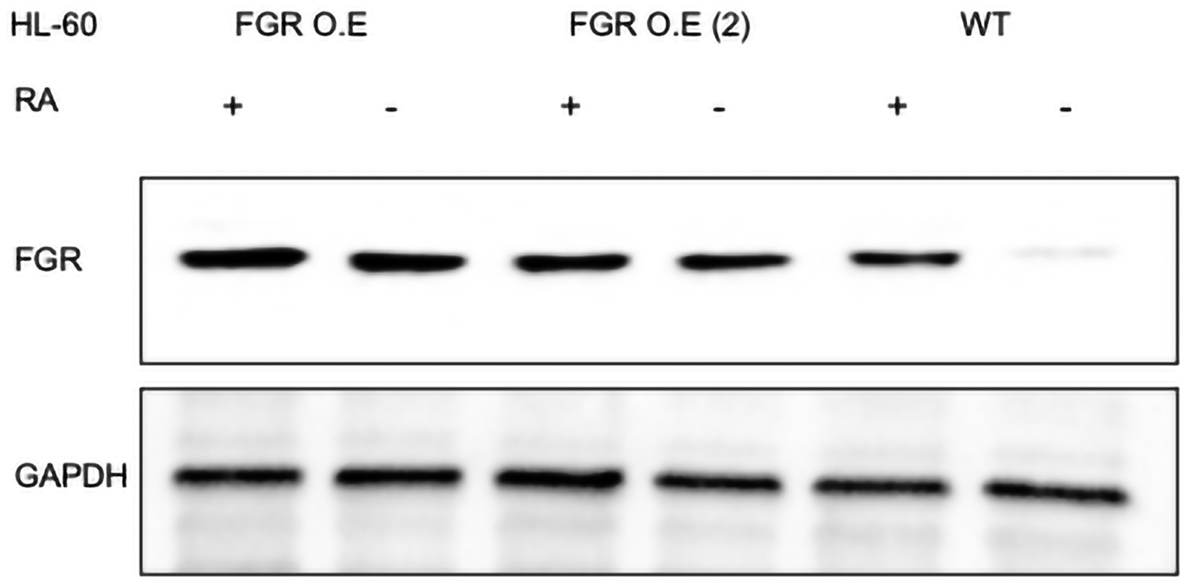

Supplementary Figure 1: FGR Western blot analysis of HL-60 wt and FGR O.E cells untreated and treated with RA. Wild-type and Fgr O.E HL-60 cells were untreated (control) or treated for 72 h with RA (1 μM) as indicated. 25 μg of lysate per lane was resolved by SDS PAGE and electro- transferred to membranes. Membrane images for each protein are cropped to show only the band of interest. This second FGR stable transfectant was created using a lenti viral expression vector. Lenti 293T cells were seeded in 6-well plate at 70–80% confluency. On the following day, 500 uL serum free medium contains Lenti-FGR plasmid, lentivirus envelope and package plasmids pMD2.G (Addgene, #12259) and psPAX2 (Addgene, #12260) with a ratio of 2 μg : 1 μg : 2 μg was mixed with another 500 uL serum free medium contains 15 ug PEI, the mixture was allowed to stay at room temperature for 15 min; Then, the cell culture medium was replaced with the mixture above, and after incubating in the incubator for 2 h, changed with 2 mL fresh medium. After 24–30 h, the supernatant containing virus was collected, 1 mL virus was added into 4 mL (density 0.1×10^6 ml) HL-60 cells with 8 μg/mL polybrene and incubated for 6 h. Then, replacing medium with fresh 1640 medium and allowing cells growth for another 24 h. Cells were then selected in the medium containing 1ug/ml puro. This transfection method is in contrast to the original FGR stable transfectant; indicating that effects of FGR transfection were ergo not vector specific/dependent.

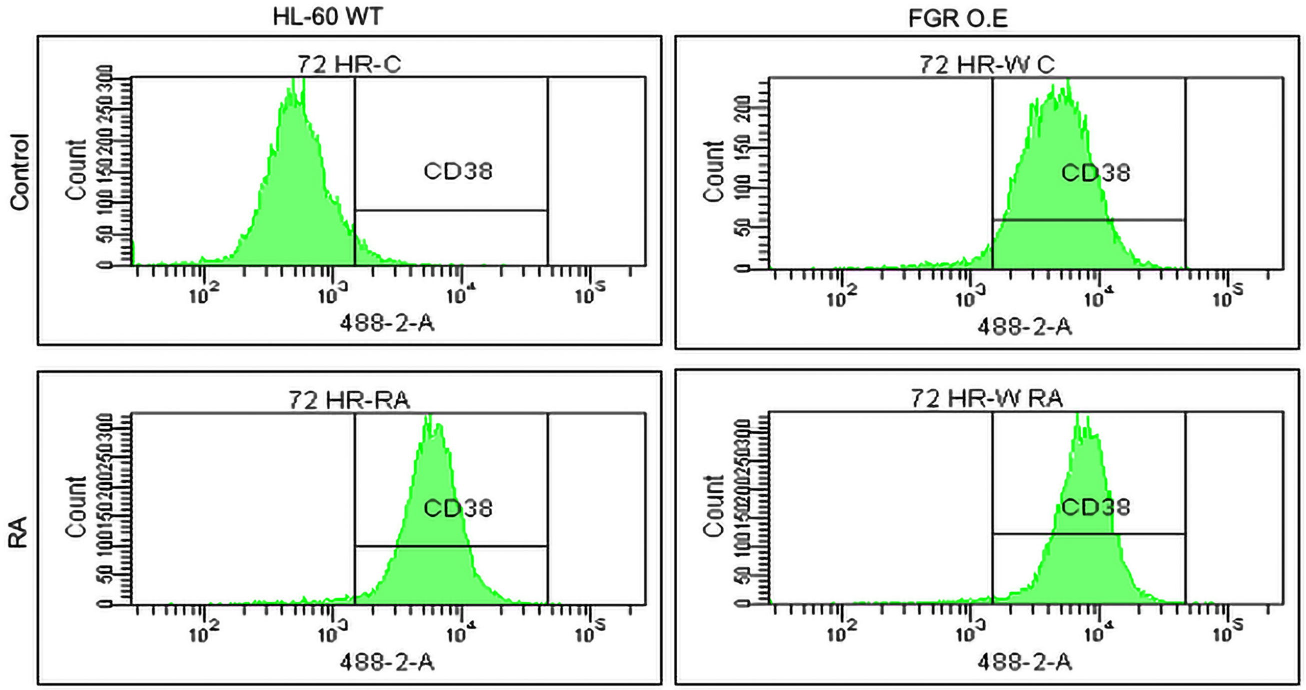

Supplementary Figure 2: Phenotypic cell surface differentiation marker analysis of HL-60 wt and FGR O.E cells untreated and treated with RA. HL-60 cells were cultured in the absence (control) or presence of 1 μM RA as indicated. CD38 expression was assessed by flow cytometry following 72 h treatment period. Gating to discriminate positive cells was set to exclude 95% of untreated controls.

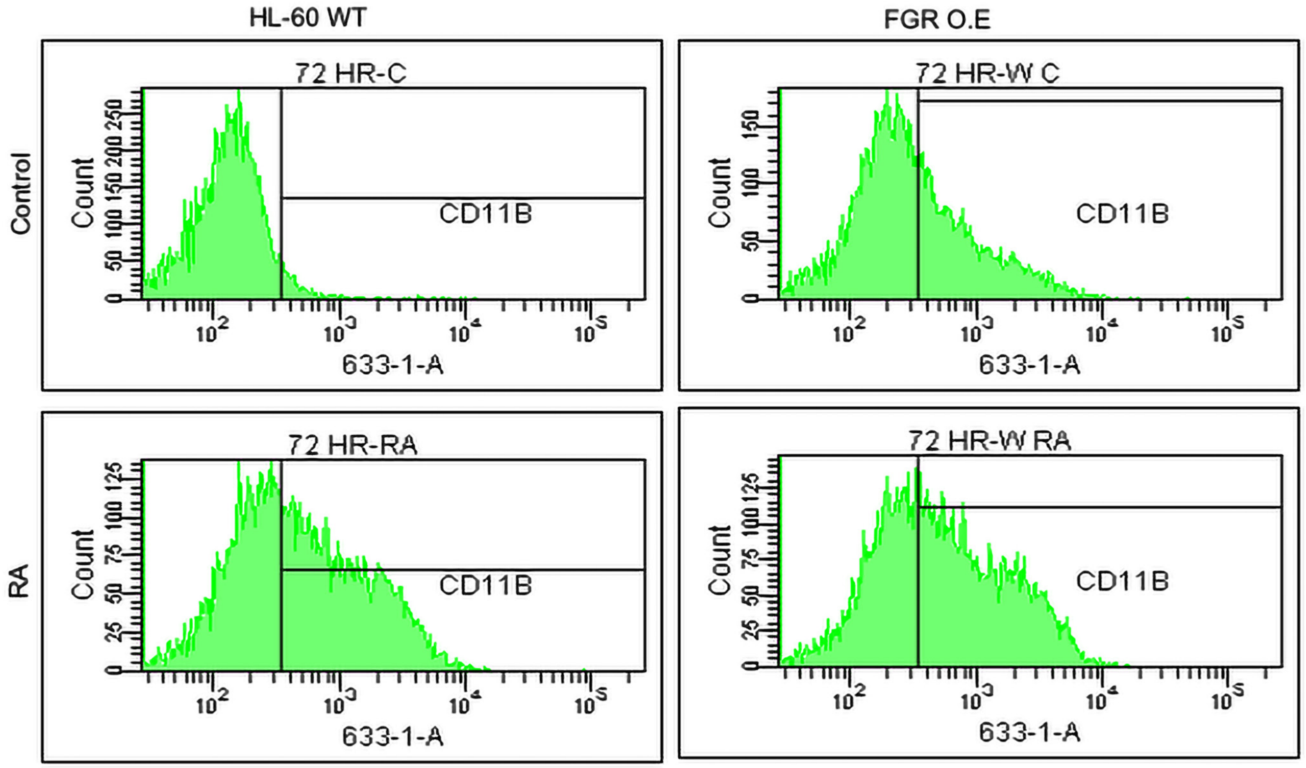

Supplementary Figure 3: Phenotypic cell surface differentiation marker analysis of HL-60 wt and FGR O.E cells untreated and treated with RA. CD11b expression was assessed by flow cytometry after 72 h treatment periods Gating to discriminate positive cells was set to exclude 95% of untreated controls.

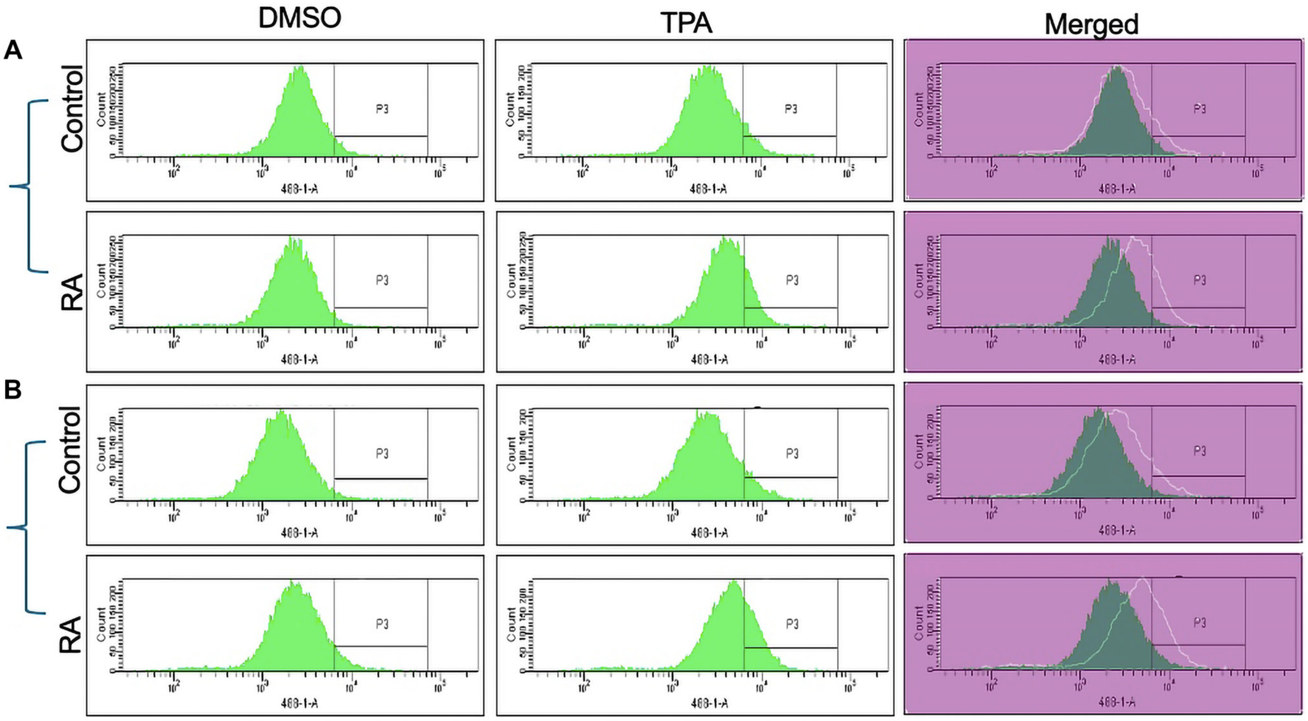

Supplementary Figure 4: Functional differentiation marker analysis of HL-60 wt and FGR O.E cells untreated and treated with RA measured by TPA-induced respiratory burst. (**A**) HL-60 WT (parental wildtype) cells were cultured in the absence (control) or presence of 1 μM RA as indicated. (**B**) FGR O.E cells were cultured in the absence or presence of 1 μM RA as indicated. Respiratory burst was analyzed by measuring reactive oxygen species (ROS) production by flow cytometry using the 2′,7′-dichlorofluorescein (DCF) assay for DMSO carrier control and TPA induced cells. Gates shown in the histograms were set to exclude 95% of the DMSO-treated control population (carrier control) for each culture condition. For each of the 4 cases, WT and FGR that were control and RA-treated, TPA-treated samples show induced ROS. Inducible ROS production is betrayed by the shift in the TPA histogram compared to the DMSO histogram shown in the merged histogram.

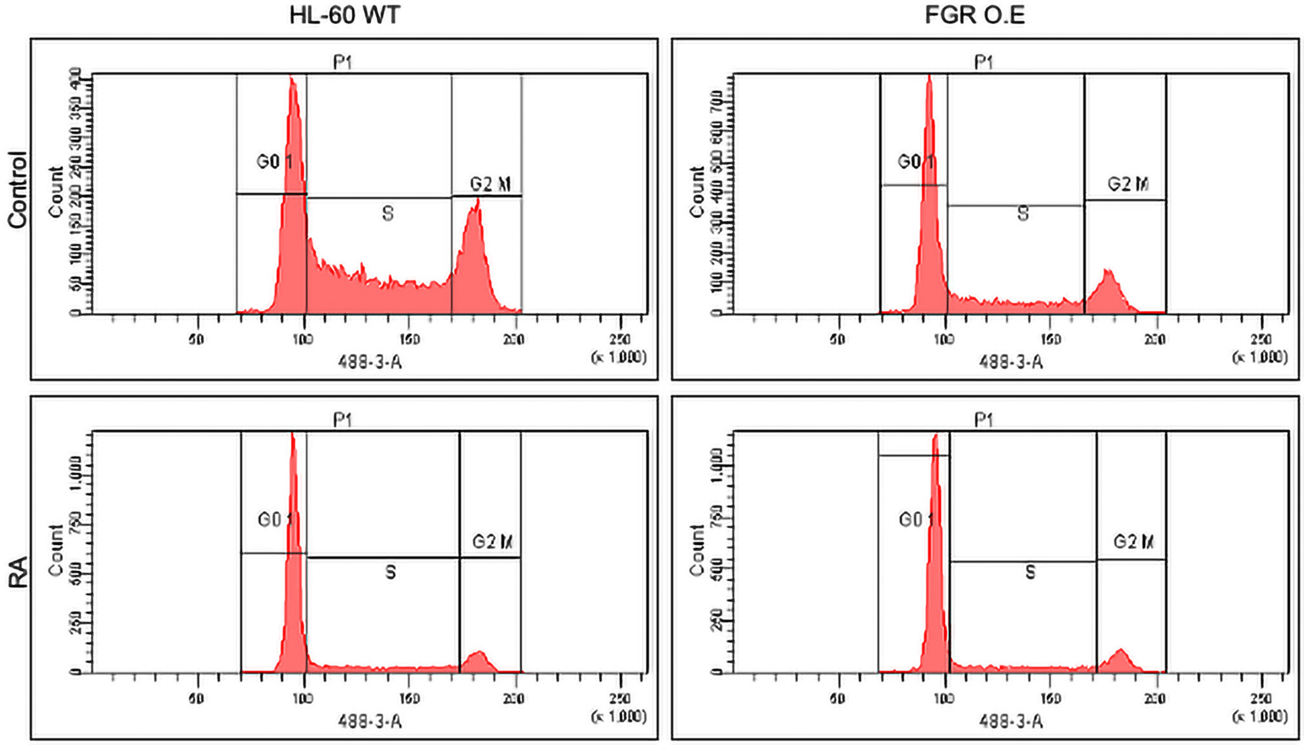

Supplementary Figure 5: Cell cycle analysis of HL-60 WT and FGR O.E cells. DNA histograms show FGR transfectants were enriched for relative number of G1/0 cells compared to the wt cells. Wild- type and FGR O.E cell lines were cultured for 72 h without (untreated control) or with 1 µM RA as indicated. Cell cycle distribution showing the percentage of cells in G1/G0 was analyzed using flow cytometry with propidium iodide staining at 72 h. Gates define the G1, S, and G2/M subpopulations (left to right). G1/0 arrest is indicated by an increase in the G1 peak for FGR O.E compared to wt cells. The G1/0 enrichment of FGR stable transfectants compared to WT cells is associated with slower population growth of these FGR stable transfectants, as well as the original transfectants, as expected. Histogram shows percentage of cells in each phase.

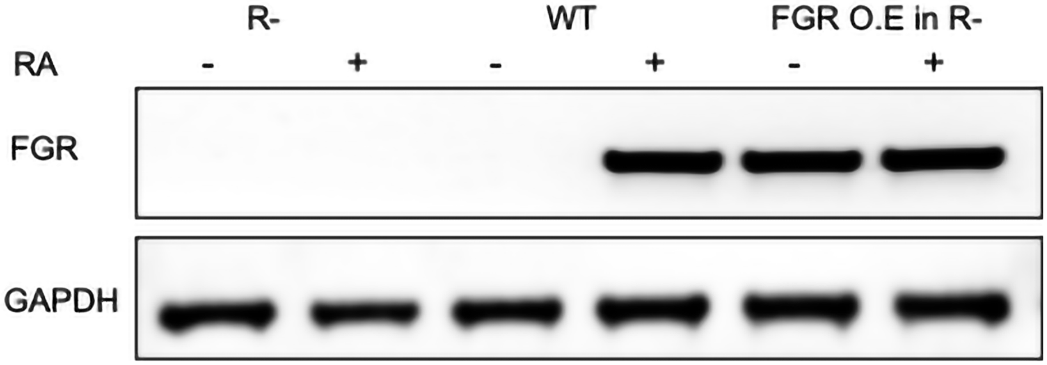

Supplementary Figure 6: FGR Western blot analysis of HL-60 WT, HL-60 R- resistant cells and FGR O.E in R- cells untreated and treated with RA. Wild-type and FGR O.E HL-60 cells were untreated (control) or treated for 72 h with RA (1 µM) as indicated. 25 µg of lysate per lane was resolved by SDS PAGE and electro-transferred to membranes. Membrane images for each protein are cropped to show only the band of interest.

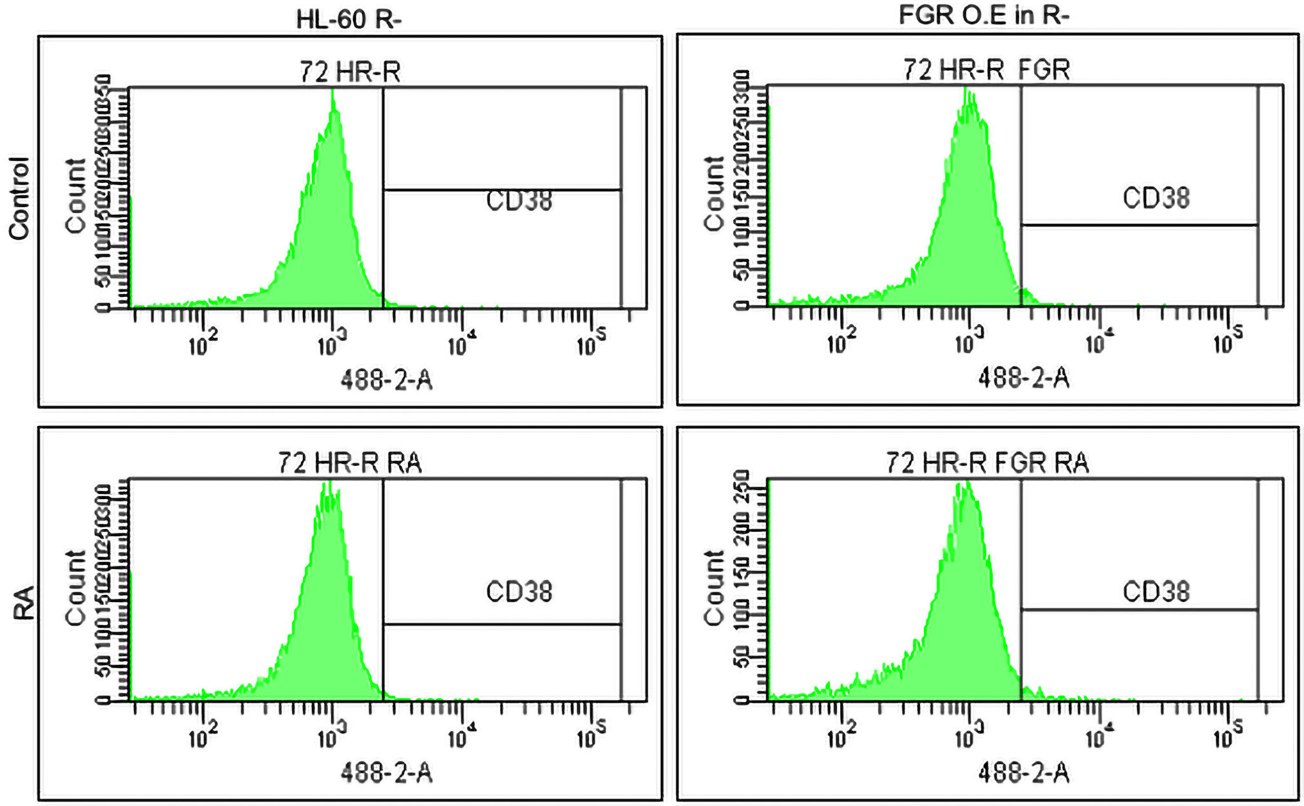

Supplementary Figure 7: Phenotypic cell surface differentiation marker analysis of HL-60 R- resistant cells and FGR O.E in R- cells untreated and treated with RA. HL-60 cells were cultured in the absence (control) or presence of 1 μM RA as indicated. CD38 expression was assessed by flow cytometry following 72 h treatment period. Gating to discriminate positive cells was set to exclude 95% of untreated controls.

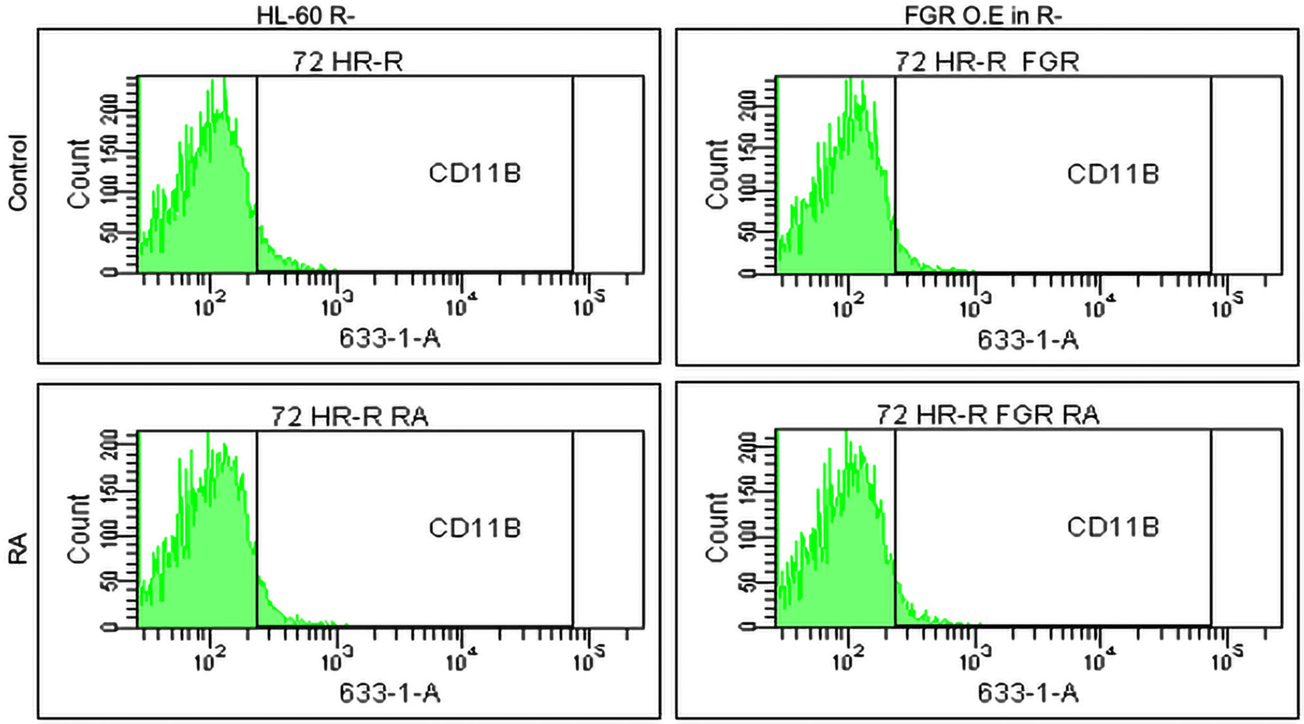

Supplementary Figure 8: Phenotypic cell surface differentiation marker analysis of HL-60 R- resistant cells and FGR O.E in R- cells untreated and treated with RA. CD11b expression was assessed by flow cytometry after 72 h treatment periods Gating to discriminate positive cells was set to exclude 95% of untreated controls.

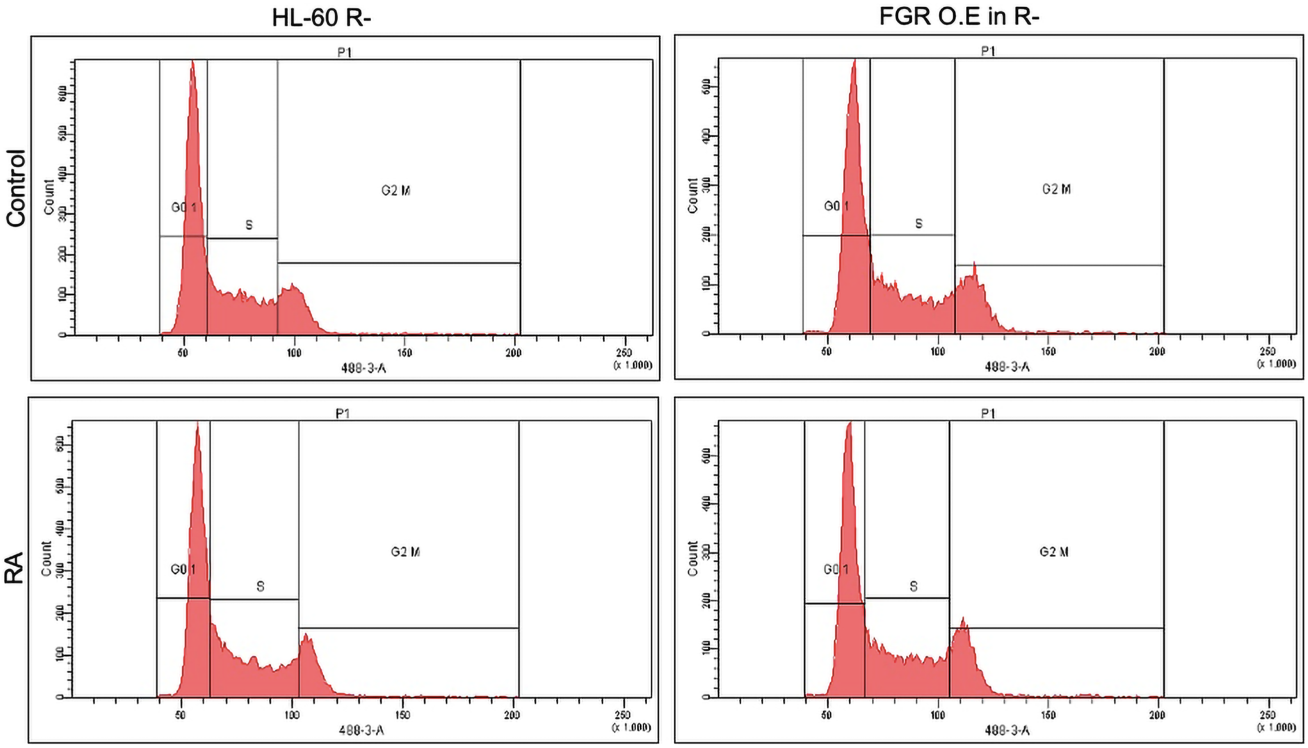

Supplementary Figure 9: Cell cycle analysis of HL-60 R- resistant cells and FGR O.E in R- cells. HL-60 R- and FGR O.E in R- cell lines were cultured for 72 h without (untreated control) or with 1 μM RA as indicated. Cell cycle distribution showing the percentage of cells in G1/G0 was analyzed using flow cytometry with propidium iodide staining at 72 h. Gates define the G1, S, and G2/M subpopulations (left to right). Lack of G1/0 arrest is indicated by a lack of increase in the G1 peak for HL-60 R- and FGR O.E in R- control and RA treated cells.

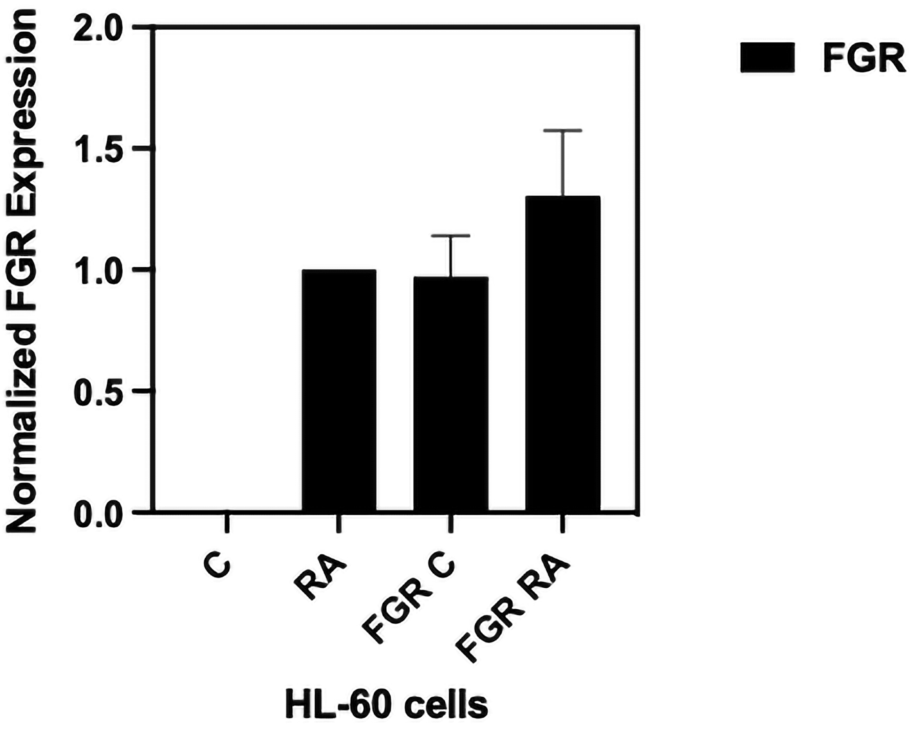

Supplementary Figure 10: The FGR histogram shows normalized densitometric values of the FGR western blot analysis of HL-60 Wt and FGR O.E cells untreated and treated with RA. ($n = 3$). Error bars indicate SEM.

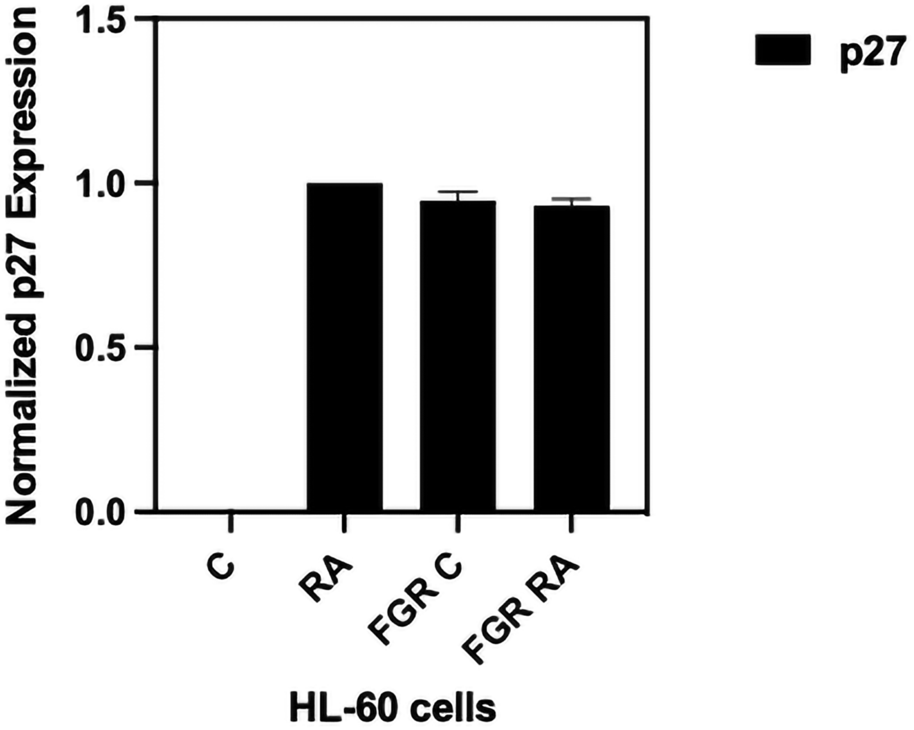

Supplementary Figure 11: The p27 histogram shows normalized densitometric values of the p27 western blot analysis of HL-60 Wt and FGR O.E cells untreated and treated with RA. (*n* = 3). Error bars indicate SEM.

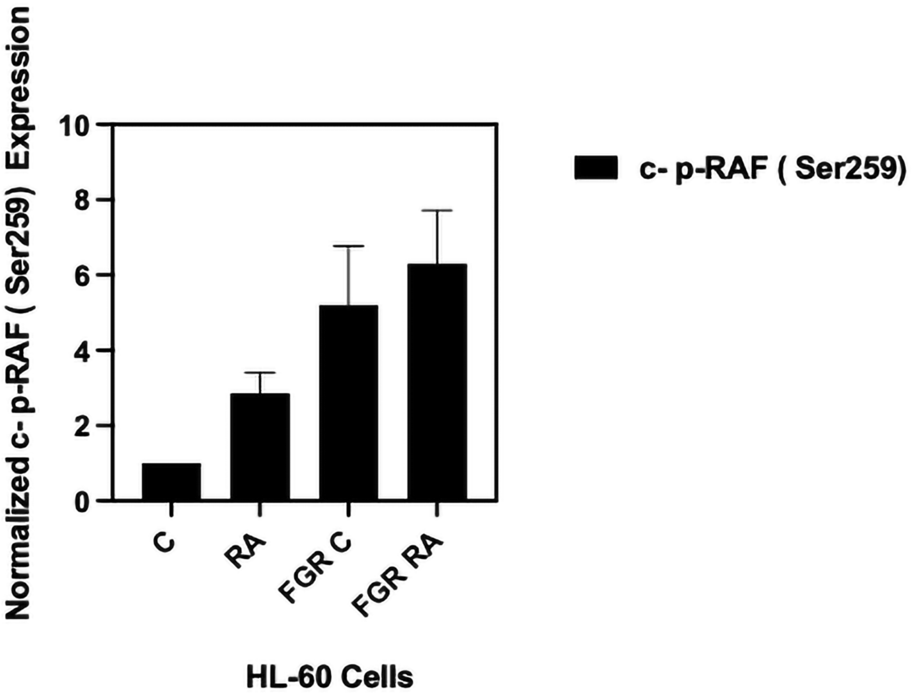

Supplementary Figure 12: p-c-RAF (Ser259) histogram shows normalized densitometric values of the p-c-RAF (Ser259) western blot analysis of HL-60 wt and FGR O.E cells untreated and treated with RA. (*n* = 3). Error bars indicate SEM.

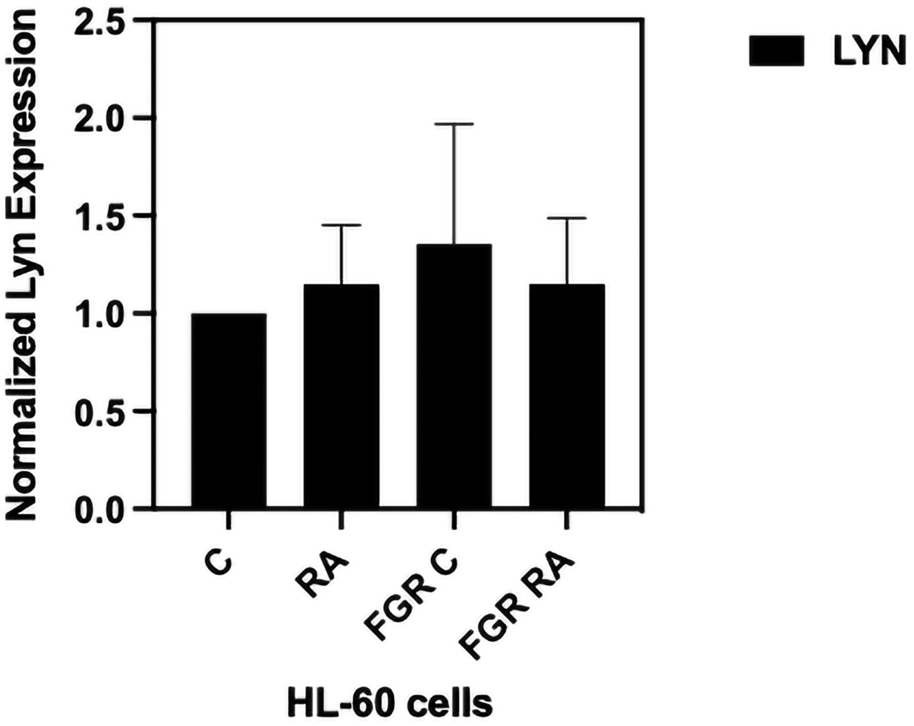

Supplementary Figure 13: The LYN histogram shows normalized densitometric values of the LYN western blot analysis of HL-60 wt and FGR O.E cells untreated and treated with RA. (*n* = 3). Error bars indicate SEM.

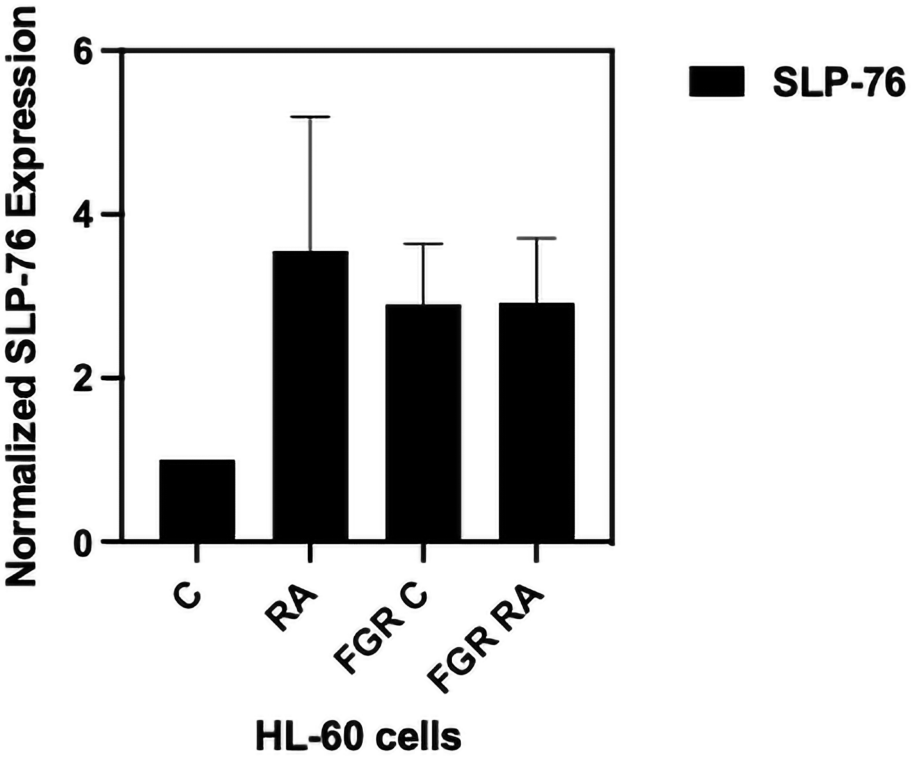

Supplementary Figure 14: The SLP-76 histogram shows normalized densitometric values of the SLP-76 western blot analysis of HL-60 wt and FGR O.E cells untreated and treated with RA. (*n* = 3). Error bars indicate SEM.

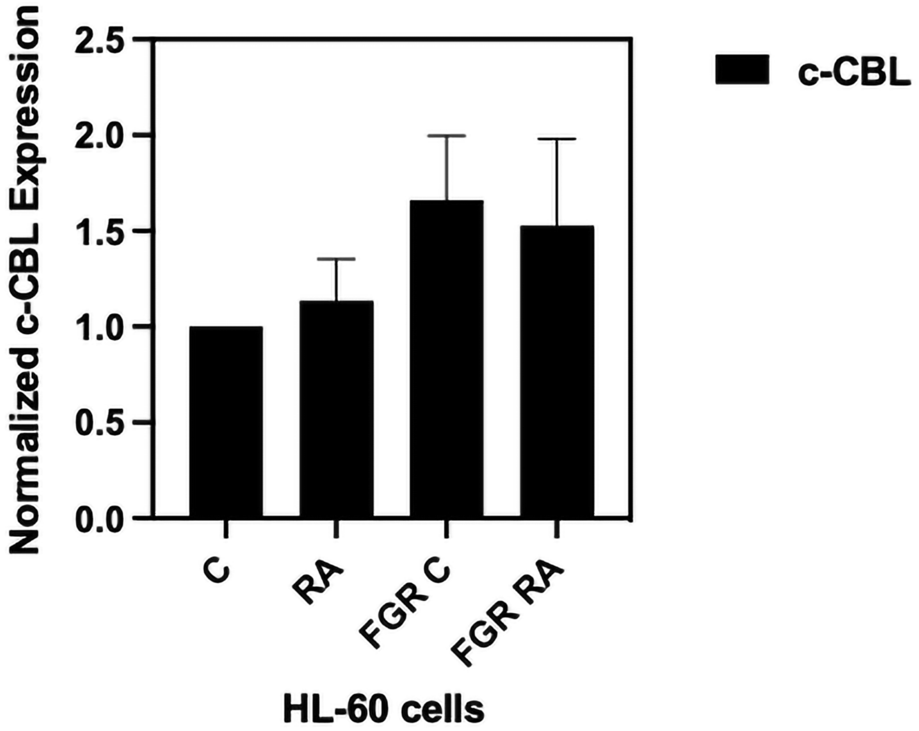

Supplementary Figure 15: The c-CBL histogram shows normalized densitometric values of the c-CBL western blot analysis of HL-60 wt and FGR O.E cells untreated and treated with RA. (n = 3). Error bars indicate SEM.

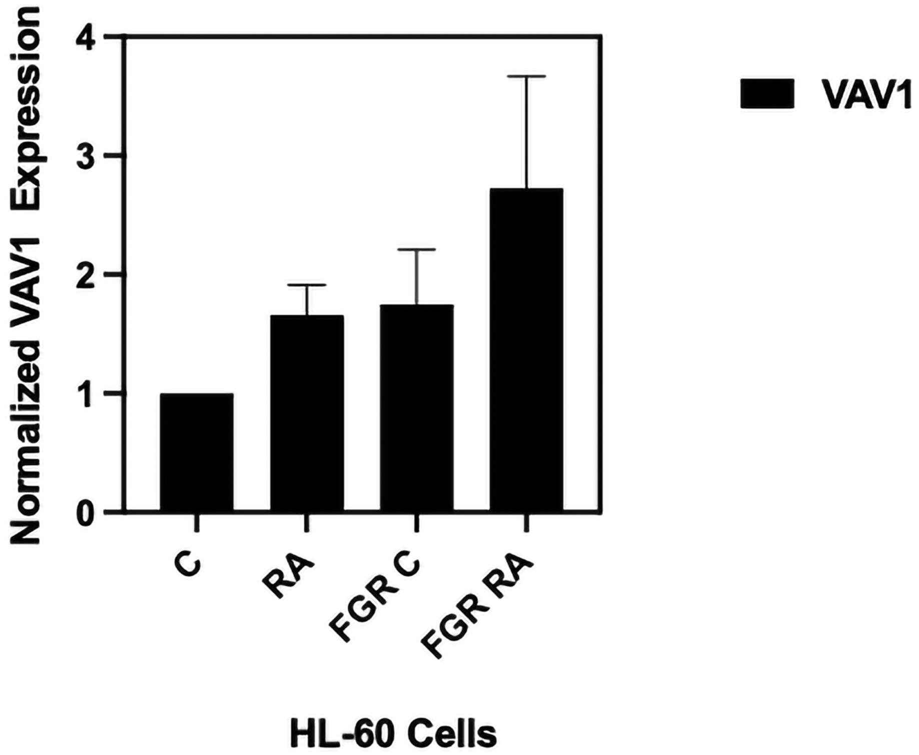

Supplementary Figure 16: The VAV1 histogram shows normalized densitometric values of the VAV1 western blot analysis of HL-60 wt and FGR O.E cells untreated and treated with RA. (n = 3). Error bars indicate SEM.

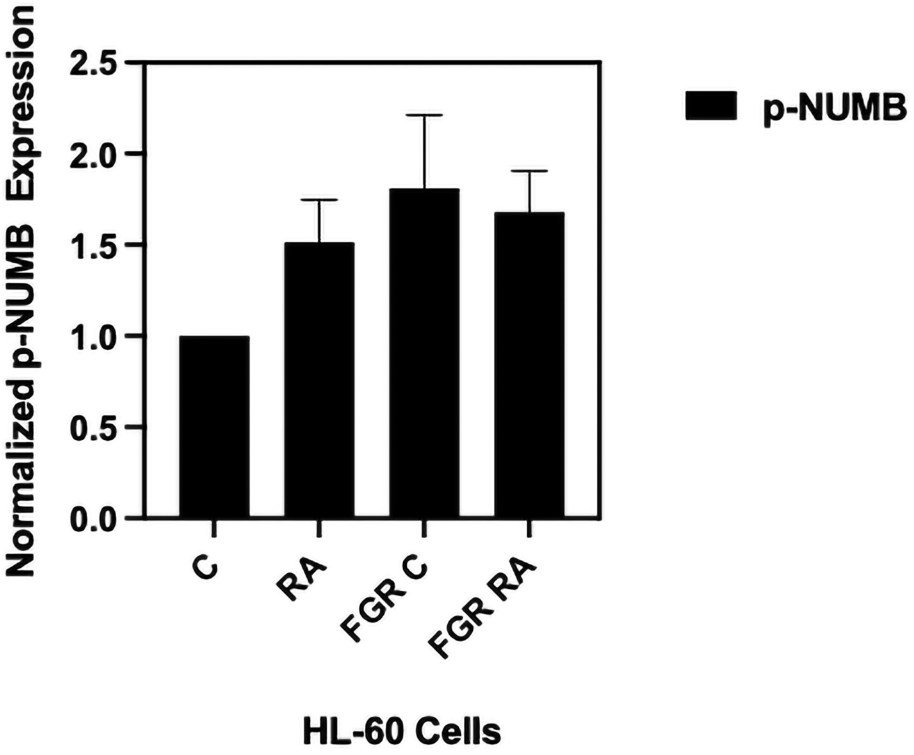

Supplementary Figure 17: The p-Numb histogram shows normalized densitometric values of the p-Numb western blot analysis of HL-60 wt and FGR O.E cells untreated and treated with RA. (*n* = 3). Error bars indicate SEM.

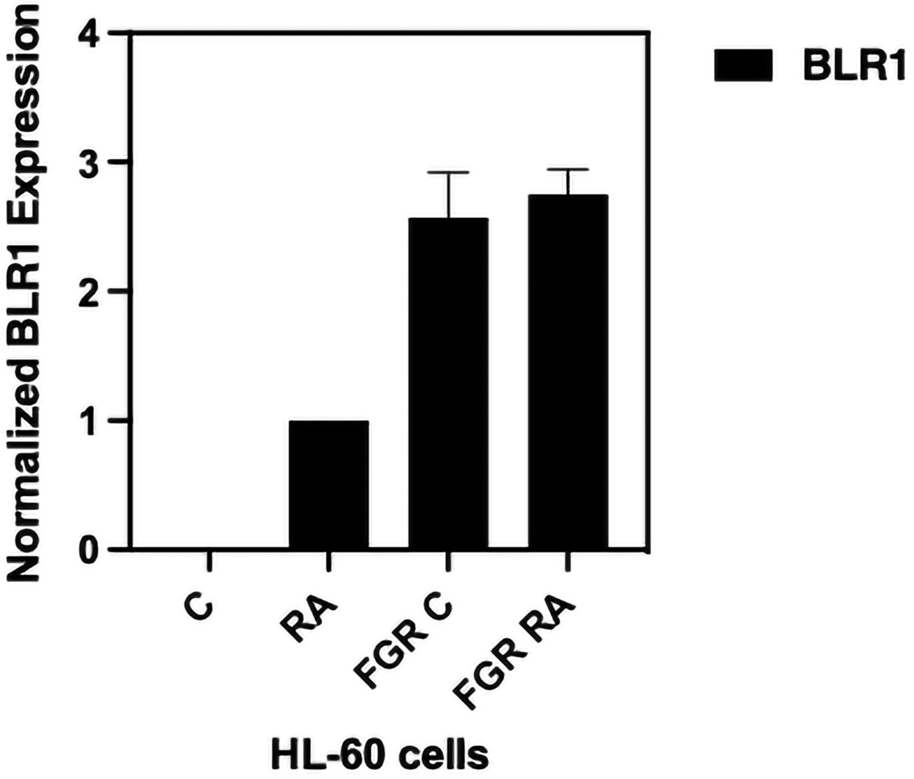

Supplementary Figure 18: The BLR1 histogram shows normalized densitometric values of the BLR1 western blot analysis of HL-60 wt and FGR O.E cells untreated and treated with RA. (*n* = 3). Error bars indicate SEM.

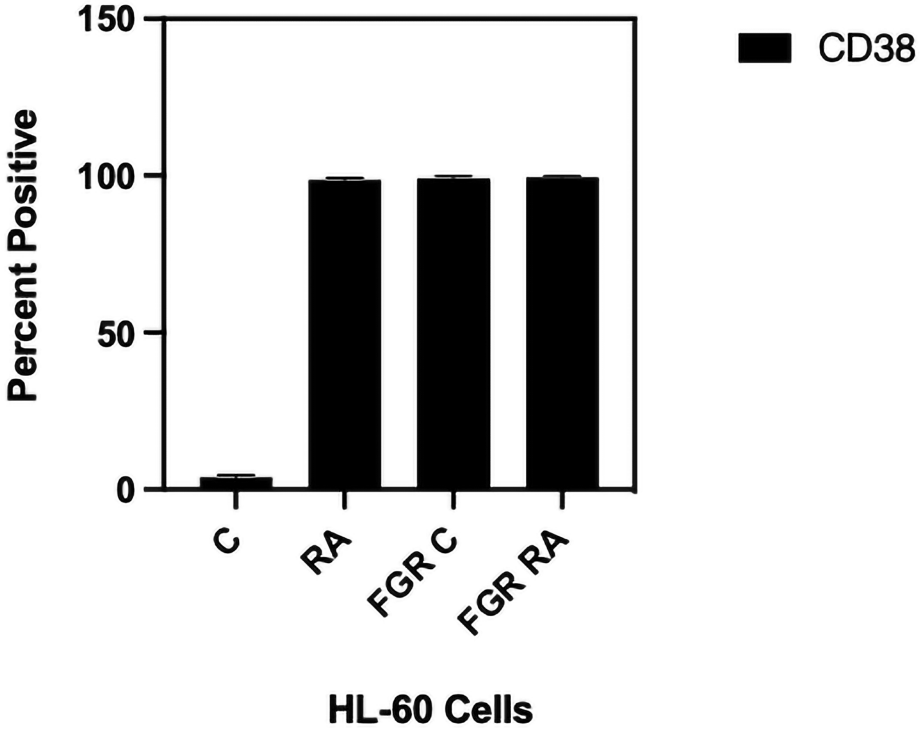

Supplementary Figure 19: The CD38 expression histogram Percentage of cells positive for CD38 expression at 72 h (*n* = 3). Error bars indicate SEM comparing RA-treated HL-60 wt samples to RA FGR O.E cells samples.

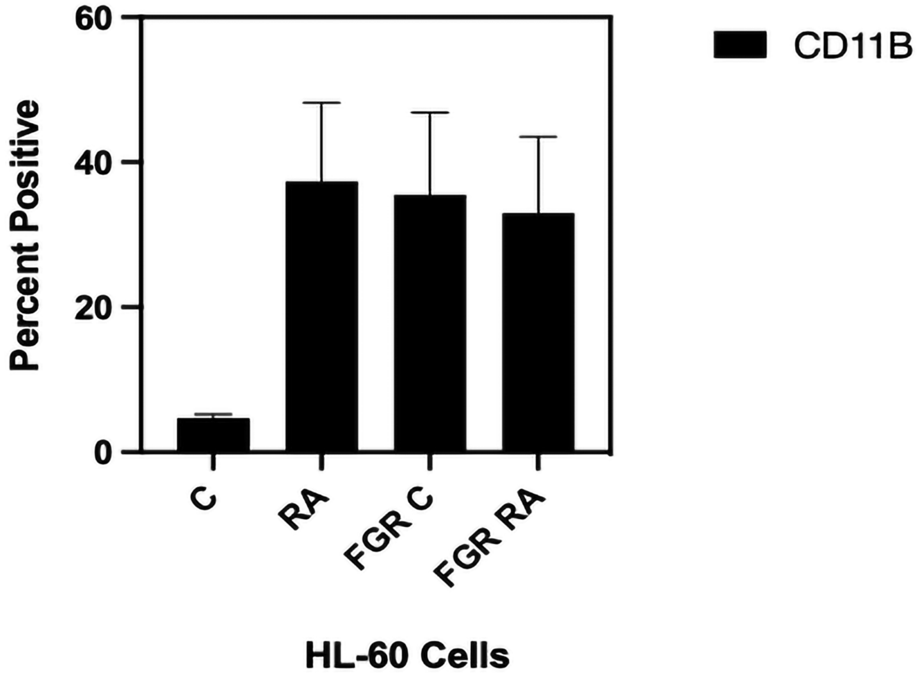

Supplementary Figure 20: The CD11B expression histogram Percentage of cells positive for CD11B expression at 72 h (*n* = 3). Error bars indicate SEM comparing RA-treated HL-60 wt samples to RA FGR O.E cells samples.

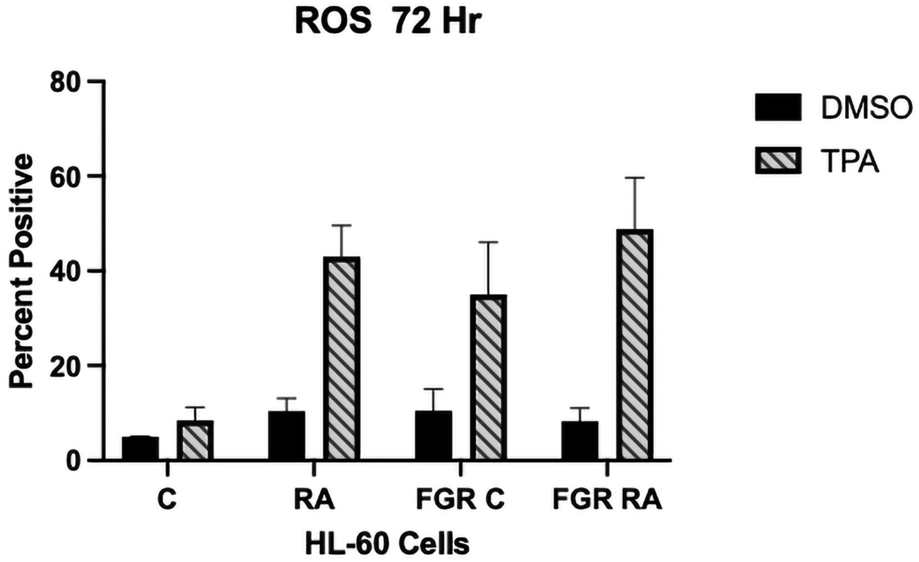

Supplementary Figure 21: Functional differentiation marker analysis of HL-60 wt and FGR O.E cells untreated and treated with RA measured by TPA-induced respiratory burst. HL-60 WT (parental wildtype) cells were cultured in the absence (control) or presence of 1 μM RA as indicated. FGR O.E cells were cultured in the absence or presence of 1 μM RA as indicated. Respiratory burst was analyzed by measuring reactive oxygen species (ROS) production by flow cytometry using the 2′,7′-dichlorofluorescein (DCF) assay for DMSO carrier control and TPA induced cells. For each of the 4 cases, WT and FGR that were control and RA-treated, TPA- treated samples show induced ROS (*n* = 3). Error bars indicate SEM.

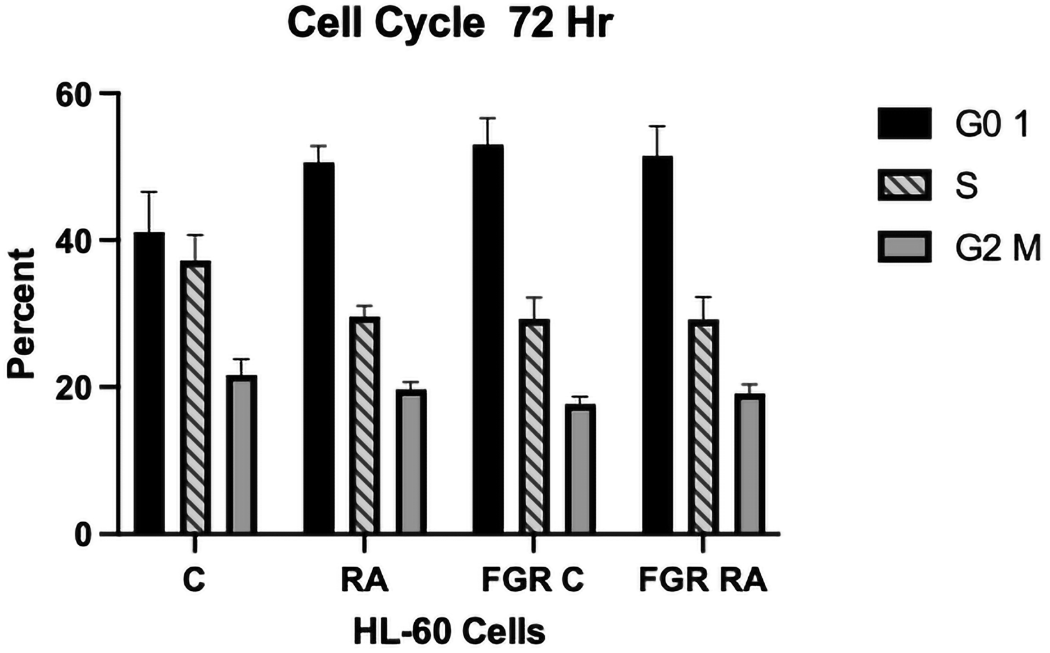

Supplementary Figure 22: Cell cycle analysis of HL-60 WT and FGR O.E cells. Histogram shows percentage of cells in each phase. Error bars indicate SEM (*n* = 3) comparing untreated samples to RA treated samples.
